# Introduction to the Special Collection: Person‐Centered Care Planning for Persons With Multiple Chronic Conditions

**DOI:** 10.1002/lrh2.70112

**Published:** 2026-07-28

**Authors:** David A. Dorr, Ana R. Quiñones

**Affiliations:** ^1^ Division of Informatics, Clinical Epidemiology and Translational Data Science, Department of Medicine Oregon Health & Science University Portland Oregon USA; ^2^ Department of Family Medicine Oregon Health & Science University Portland Oregon USA; ^3^ OHSU‐PSU School of Public Health Oregon Health & Science University Portland Oregon USA

Multiple chronic conditions (MCC) affect approximately 130 million American adults, representing a critical health care challenge that standard, fragmented disease management has failed to adequately resolve. Evidence increasingly supports person‐centered care planning (PCCP)—defined as a longitudinal care approach that places patient goals, values, and preferences at the center of treatment—as a necessary standard of practice. To address the gaps in current approaches and care models for management of MCC, this special collection presents four manuscripts emerging from a multi‐faceted initiative to advance PCCP for those with or at risk for MCC, supported collectively by funding from the Agency for Healthcare Research and Quality (AHRQ), the John A. Hartford Foundation, and the Patient‐Centered Outcomes Research Institute (PCORI). Together, these papers evaluate the current evidence base, operational challenges, on‐the‐ground clinician and patient experiences, and the evidence‐based integration of symptom management into primary care.

The collection opens with foundational work by Totten, Dorr, Bierman, and colleagues, who conducted an environmental scan that informed the broader AHRQ initiative. Through a rigorous multi‐component review of published and gray literature, the authors identified 40 PCCP models currently in use across diverse US health care settings. These models share a common commitment to whole‐person care but differ substantially in how they achieve it—through making changes in the way care is provided. The authors characterized this change in care as focused on team roles and composition, technology, payment structures, clinical functions, and care focus. Approximately two‐thirds of evaluated models demonstrated better or equivalent outcomes compared with usual care. The scan was complemented by qualitative interviews with 18 key informants who were able to provide rich contextual details on what works, how approaches can be improved, and what is impeding broadscale change. Barriers to adoption were consistent across settings: insufficient time, misaligned payment structures, and the cultural challenge of reorienting care delivery from disease management to patient‐defined goals. Facilitators included flexible team structures, alignment with organizational mission, and leadership support. The environmental scan makes clear that a strong evidence base exists—what is lacking is the infrastructure, incentives, and implementation support to bring these models to scale.

The summary article by Davis, Coury, Nagykaldi, and colleagues presents findings from the PCCP for Persons with MCC Summit, a culminating event convened at AHRQ headquarters in Rockville, Maryland in March 2025. Drawing on 14 months of prior work, the Summit brought together 115 participants across sectors, including researchers, payers, health system leaders, community organizations, patients, and policymakers. Three broad takeaways emerged as essential to the goal of advancing PCCP: improving patient experience, strengthening the clinician‐patient relationship, and reducing care team burnout. Summit participants identified six interconnected findings to encourage PCCP and make it routine practice: demand and awareness for this kind of care, quality measurement, team‐based care, technology, payment reform, and workforce training; and generated actionable implementation steps across sectors. The manuscript emphasizes that scaling PCCP will require coordinated, multi‐sector efforts and identifies implementation science and learning health system approaches as critical enabling tools to achieve these ends.

The manuscript by Henningfield, Sachs, Schrager and colleagues, shifts the lens to the patient experience—one of the areas the AHRQ initiative sought to advance. Presented as a commentary drawing on patient narratives developed for and presented at the AHRQ Summit, the manuscript centers two first‐person accounts that illustrate what happens when care planning excludes the patient's voice. The first narrative, from a patient managing 17 diagnoses, documents the downstream consequences of a paternalistic care model—non‐adherence, iatrogenic harm—and contrasts with improved care and wellbeing through engagement with a clinician who finally invited the patient into shared decision‐making: “I drove and he navigated until I needed a break. Then he drove and we chose the route together.” A second narrative details how the absence of a coordinated, person‐centered discharge plan—compounded by insurance barriers, inaccessible transportation, and inadequate follow‐up—cascades into avoidable emergency department use and eroding trust. Taken together, these narratives argue that patient engagement is not merely a value‐add to PCCP. Without it, no measure of policy change, payment incentives, or technological enablers will be effective in improving the quality of care and outcomes patients experience. The commentary concludes with a call for future work that places patients, care partners, and families as co‐designers of PCCP models, not solely as passive recipients.

The manuscript by Kuebler, Bruera, Sorenson, and Gallegos, elevates the important challenges of addressing significant and often distressing symptom burden that people with MCC experience within the realities facing primary care. Drawing on a narrative synthesis of major policy recommendations, clinical guidelines, and the palliative care literature, the authors propose a practical, stepped framework for integrating palliative symptom management into routine primary care. The framework uses the Edmonton Symptom Assessment System (ESAS)—a validated, freely available, multilingual tool—as a universal screening instrument and structures escalation from primary care‐led symptom management through consultative support to specialty outpatient palliative care based on symptom complexity and functional trajectory rather than prognosis alone. The manuscript is notable for its insistence that palliative care belongs upstream in the disease course for people with MCC—not reserved solely for end‐of‐life care—and that primary care teams, with targeted training and protocolized tools, are capable of delivering it. This reframing of palliative care as a core competency of primary care practice directly supports the PCCP goals that the collection advances as a whole.

These four manuscripts collectively demonstrate that PCCP for individuals with MCC is an evidence‐backed and highly achievable standard of care, rather than a distant aspiration. By highlighting adaptable clinical models, accessible tools, and the critical necessity of patient engagement, the collection provides a clear, actionable blueprint for systemic impact. The overarching conclusion is that broadscale implementation now relies on the collective will of health systems, payers, clinicians, patients, care partners, researchers, and policy makers to establish the foundational infrastructure, workforce training, and payment alignment necessary to make person‐centered care a sustainable reality for the 130 million Americans who require it.

## Funding

This project was funded under Contract No. 75Q80120D00019/75Q80124F32002 from the Agency for Healthcare Research and Quality (AHRQ), U.S. Department of Health and Human Services (HHS). The John A. Hartford Foundation also provided funding to support project dissemination.

## Data Availability

Data sharing is not applicable to this article as no datasets were generated or analysed during the current study.

